# Changes in the Review Period of Drug Application and a Drug Lag from the FDA and the EMA: An Industry Survey in South Korea Between 2011 and 2020

**DOI:** 10.1007/s43441-022-00486-x

**Published:** 2022-12-20

**Authors:** Hyeyoung Choi, Hyesung Lee, Bojung Park, Chorong Kim, Jaehyun Lee

**Affiliations:** 1 School of Pharmacy, Sungkyunkwan University, Suwon, South Korea; 2 Department of Biohealth Regulatory Science, Sungkyunkwan University, Suwon, South Korea; 3 Korean Research-Based Pharmaceutical Industry Association, Regulatory Affairs, Seoul, South Korea

**Keywords:** New drug application, Drug approval, Drug lag, Review period, Submission

## Abstract

**Background:**

The Korean regulatory authority has enacted legislation to expedite the new drug approval (NDA) process. However, the effectiveness of such efforts in reducing review time and drug approval delays between Korea and the USA/EU remains to be evaluated.

**Methods:**

We investigated NDA trends in Korea from 2011 to 2020 using approval information from pharmaceutical companies. We compared the changes in the actual review duration according to active ingredient (chemical vs. biological), orphan status, therapeutic class, and NDA review process. We estimated the submission and approval gaps of new drugs between Korea and the US and EU across the study period.

**Results:**

For 235 new drugs, the median NDA review time was 315 days, with a significant increase in the delay (average 15.4 days) over time. Biological drugs had a 43.2-day delay for approval than the time taken for approving chemical drugs. The median NDA review time for orphan drugs was 130.4 days faster than that for others, although the difference diminished after 2016. Good manufacturing practice reviews played a crucial role in delaying review time. The median submission and approval gaps in Korea were 493 and 551 days, respectively, compared to those of the US and EU.

**Conclusions:**

Despite recent legislative initiatives, the delay in the NDA review timeline has steadily increased over 10 years in Korea. Delays in orphan drugs reviews increased after the enactment of the ‘Rare Disease Management Act’ in 2016. Careful enforcement of relevant laws and supplementary actions is required to increase new drug accessibility.

**Supplementary Information:**

The online version contains supplementary material available at 10.1007/s43441-022-00486-x.

## Introduction

The new drug approval (NDA) review period is an important factor that affects drug accessibility. Drug lag can reduce the quality of life among patients in certain geographies with limited access to important new therapies. Every year, the UK Centre for Innovation in Regulatory Science analyzes and reports data related to drug approvals from six major countries. Several countries have increased focus on reducing the time required for a new drug to be approved [1, 2]. There was a substantial difference in new drug review and approval time-frame across countries. The US FDA had the shortest median drug review time-frame (244 days), followed by Canada at 306 days, Japan PMDA at 313 days, Australia at 315 days, EU at 426 days, and Switzerland at 470 days in 2020 [1, 2]. In case of Japan, the drug review period has reduced from 367 days in 2011 to 313 days in 2020, primarily, as more PMDA consultations were conducted in Japan [1–3].

During the past decade, the Korean Ministry of Food and Drug Safety (MFDS) introduced a pre-preview and expedited review system to increase the market accessibility of new drugs and to overcome drug lag [1, 2]. The Korean MFDS amended the Pharmaceutical Affairs Act in 2011 to provide an NDA pre-review process for expedited review, while enacting and implementing NDA review guidelines to include priority review and conditional approval. Subsequently, the ‘Rare Disease Management Act’ was enacted in 2016 to set up a legal system for the expedited review of orphan drugs [4]. However, quantifying the improvement in the NDA review time-frame after implementation of the enactments is challenging due to lack of data [4].

In this study, we investigated the changes in the actual time-frame for NDA review and approval in Korea from 2011 to 2020. We identified factors affecting the review period and compared the submission and approval gaps with those of the US FDA and EMA.

## Methods

### Study Design and Data Acquisition

This study was conducted using new drugs approved in Korea by members of the Korean Research-based Pharmaceutical Industry Association (KRPIA) between 2011 and 2020. KRPIA was established in March 1999 and received official approval as an incorporated association from the Ministry of Health and Welfare in June 2000 and the MFDS in 2014. As of 2020, its membership included 45 research-oriented pharmaceutical companies. Among these, 34 companies that received approval for new drugs within the research period responded to our request. To calculate the approval review period for a new drug, we collected information such as the time of submission of original license application, approval date, ATC code classification, drug classification (chemical or biological), orphan drug status, date of supplementation data request, date of supplementary data response, good manufacturing practice (GMP) inspection date, GMP supplementation request date, and GMP review reply date. Good clinical practice (GCP) inspection is performed for the new drugs developed by domestic pharmaceutical companies, and it is included in the review period of safety efficacy assessment.

In addition, to compare the time it took for the new drugs to be approved in their initially approved country with the time the process took in Korea, the submission and approval dates of the new drugs in the FDA and EMA were also investigated.

### Study Drugs

During this period, 285 new drugs were approved in Korea [4]. However, new drugs developed by domestic pharmaceutical companies and those that did not respond to the study questionnaire were excluded from the study. Consequently, 235 new drug products were analyzed for their review periods in this study.

A dataset was established, including drug name, active pharmaceutical ingredient, company name that received the drug approval, whether it was classified as an orphan drug, WHO ATC classification [5], and domestic approval date of the new drug product [6]. The following items were then investigated and used to collect information on the time period required for approval through the MFDS’s integrated drug information system: MFDS department that received the drug application, date of application submission, date of application receipt, date of supplementary data request, date of supplementary data receipt, date of review response, whether GMP inspection or drug master file investigation was conducted and the date for investigation, date of supplementary data request, date of supplementary data receipt, date of response, whether an independent examination or preliminary examination was conducted before application, and whether the approval application was withdrawn and then re-applied [4].

### Variable Definition

The ‘review period’ was used as an outcome variable and defined as the period from when the application for approval was received by the MFDS to the issuance of the approval certificate. To analyze the factors affecting the review period of a new drug, the time required for a review process was calculated by adding the entire duration from the date of application submission to MFDS’s review response, which includes the period from the supplementary data request to the date of receipt [4].

To compare the drug lag of new drugs in Korea with that of other countries, the difference between the earliest date of application submission either in the US or Europe and the date of application submission in Korea was defined as ‘submission gap,’ and the difference between the earliest date of approval either in the US or Europe and the date of approval in Korea was defined as ‘approval gap’ [7–11].

Variables used to analyze factors affecting the NDA review period included drug classification (chemical or biological drug), orphan drug status, therapeutic area classification, GMP inspection status, and duration.

### Statistical and Data Analysis

Using a set of new drugs (*n* = 235) approved in Korea between 2011 and 2020, we conducted several analyses. First, we summarized the basic characteristics of the study drugs and review periods using frequencies with proportions and medians for Q1 and Q3. Their characteristics were divided by year, origin of the substance, orphan drug status, and therapeutic indication based on the WHO ATC code [6]. All time periods presented in the results were calculated as calendar days rather than working days. Second, the trends of the review period for the study drugs were plotted over 10 years to identify trends in the review period. Additionally, trends of the review period were stratified by the origin of substance and orphan drug status to identify the impact of the categories on the review period.

Third, multiple regression analyses were conducted to identify factors (e.g. orphan drug, biological drug, and GMP inspection) associated with the median value of the review period. In the model, the independent variables included the submission date, approval date, origin of the substance, and therapeutic class. Fourth, to examine the factors affecting the review period, the actual review period was subdivided into safety and efficacy review process, specification and test method review process, and GMP review process. Dummy variables were created for all the categorical variables. Fifth, the submission and approval gaps related to FDA or EMA approvals were examined to compare the drug approval lag between Korea and the US or Europe [7–9]. All statistical analyses were computed using SAS 9.4 (SAS Institute Inc, Cary, NC), and statistical significance was defined as *P* < 0.05.

## Results

**Figure 1:**
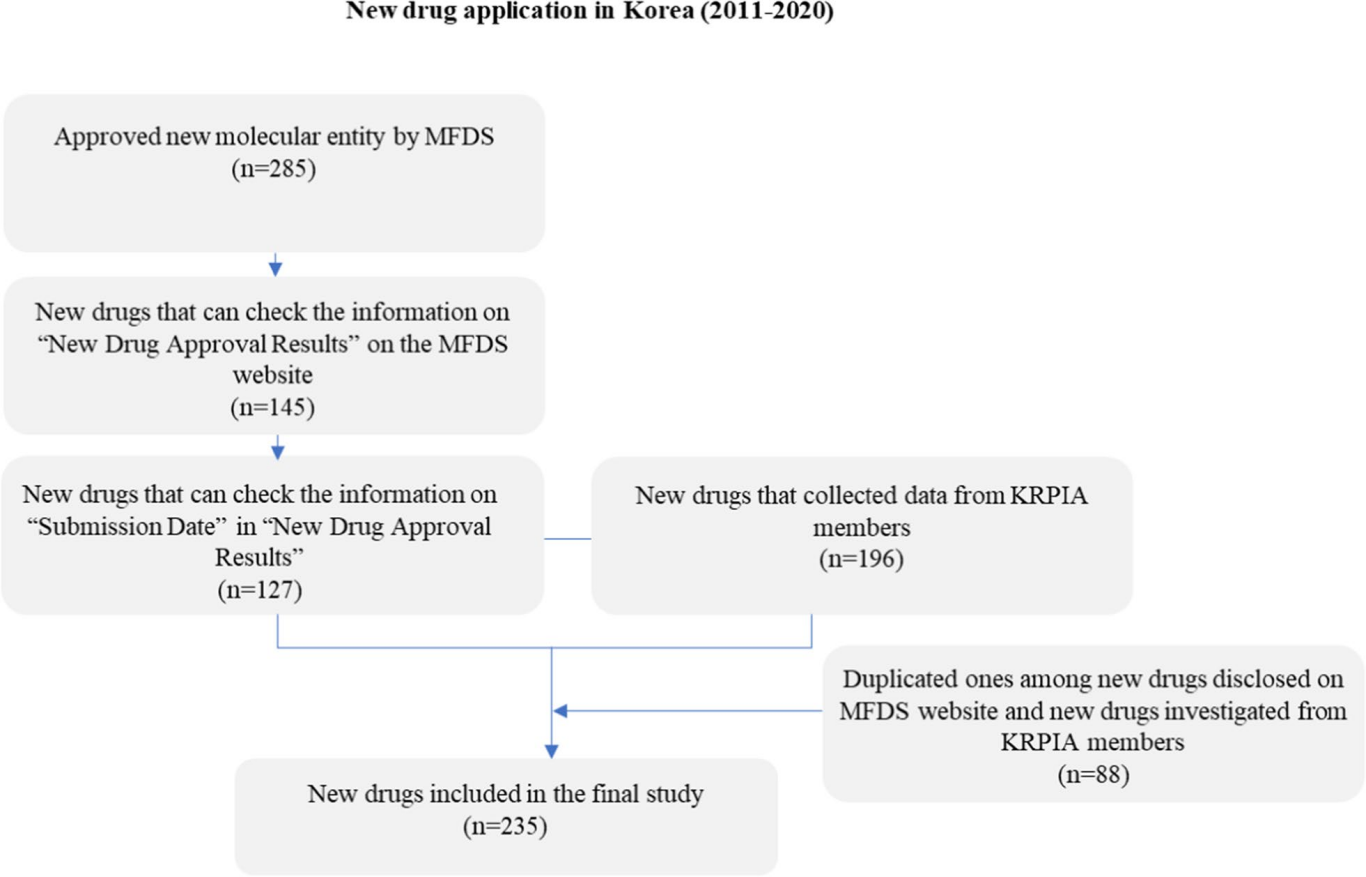
Flow diagram as study database for the selection of study drugs.

From 2011 to 2020, there were 285 new drugs approved in Korea (Fig. 1). Among them, data for 127 new drugs were published on the MFDS website and 196 were received from KRPIA. There were 88 duplicates, and the final data included in this study were 235.

The median review period of the 235 new drugs approved in Korea from 2011 to 2020 was 315 days, with an average delay of 15.4 days (*P* < 0.0001). Table 1 shows the results of the review period, which were compared and calculated based on the classification of the new drugs according to their characteristics.

**Table 1:** Characteristics of new drugs approved in Korea between 2011 and 2020.

Categories	No. of approvals (*n* = 235)		NDA timeline	Coefficient	*P* value
	n	%	Median		
Year	235	100	315	15.4	< 0.0001
*Origin of substance*					
Chemical	166	70.6	291	(Reference)	
Biologics	69	29.4	353	43.2	0.0054
*Orphan drug status*					
Standard (Reference)	147	62.6	349	(Reference)	
Orphan	88	37.4	191	− 130.4	< 0.0001
*Therapeutic indication*					
L (Anticancer and immunomodulators)	92	39.1	290	(Reference)	
A (Alimentary and metabolism)	33	14.0	302	4.9	0.8136
J (Anti-infectives)	28	11.9	304	− 34.6	0.1261
N (Nervous system)	19	8.1	331	− 17.3	0.5026
C (Cardiovascular)	10	4.3	333	4.8	0.8878
R (Respiratory system)	8	3.4	365	56.7	0.1339
G (Genito urinary system & sex hormones)	8	3.4	307	13.9	0.7263
Other TAs	37	11.5	333	26.7	0.1811
*Adjusted R*^*2*^					0.4157

Among the new drug products, 166 (70.6%) chemical drugs and 69 (29.4%) biological drugs were identified and the median time to approval was 291.0 and 353.0 days, respectively. Regression analysis showed that the approval timeline of the biological drugs was 43.2 days longer than that of chemical drugs, and the difference was statistically significant between the two groups (*P* = 0.0054).

Over the study duration, 88 orphan drugs were approved, accounting for 37.4% of all new drugs. In particular, the proportion of orphan drug approvals was high in 2015, whereas no drug was approved as an orphan drug in 2018. The approval of orphan drugs was 130.4 days faster on average than that of the other new drugs (*P* < 0.0001). The analysis also showed a difference in the review periods based on the therapeutic groups, but the difference was not statistically significant (Table 1).

### Study on NDA Review Period

**Figure 2:**
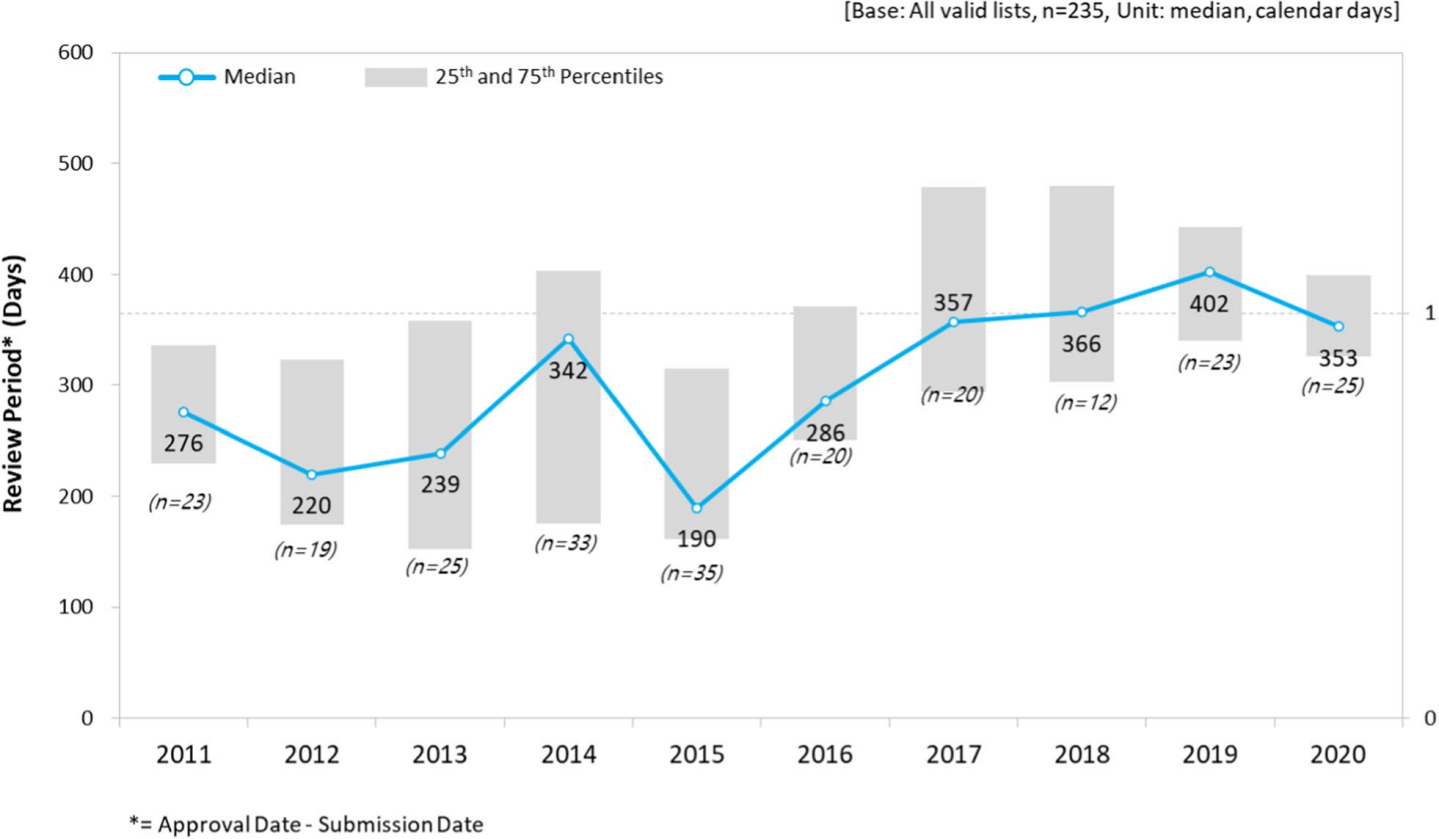
New drug application (NDA) review period.

It was not possible to confirm a certain trend in the review period of new drugs from 2011 to 2020. However, it was not clear if the overall review period actually decreased significantly. In contrast, the review period has been steadily increasing, from 276 days in 2011 to 353 days in 2020 (Fig. 2).

### Comparison of Review Period According to New Drug Characteristics

**Figure 3:**
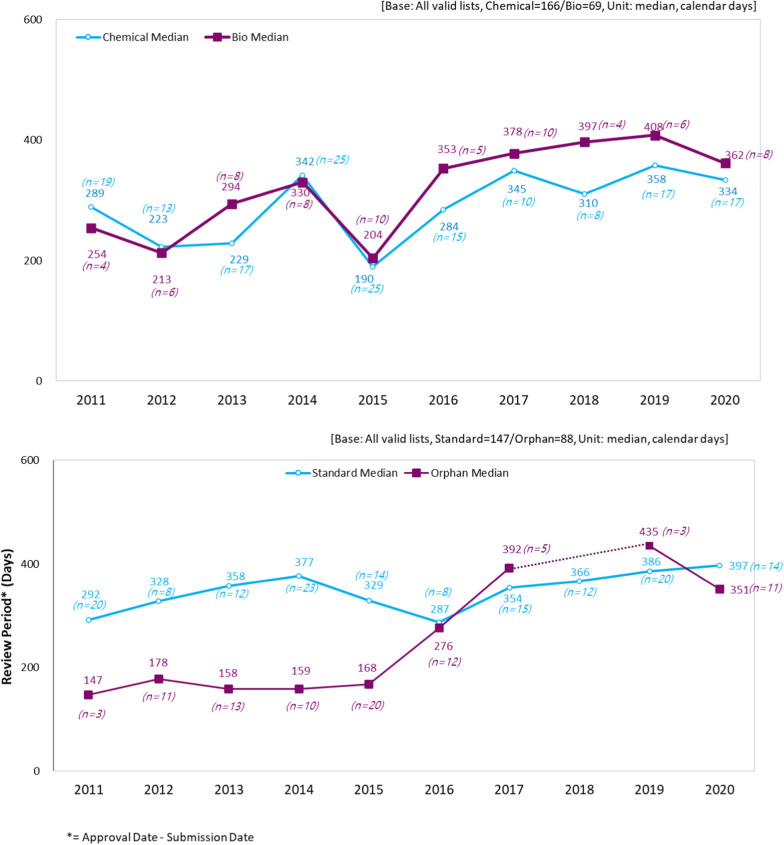
NDA review time by type of dugs (Chemical vs. Biological/Standard vs. Orphan).

There was significant difference between chemical and biological drugs during the review period (Fig. 3).

When comparing the review periods while incorporating orphan drugs, the review period for orphan drugs was twice as fast as that for a general new drug from 2011 to 2015. However, the period was similar or even longer after 2016 (Fig. 3). Similar trends were observed orphan drugs for both chemical drugs and biologics in the stratified analyses (Supplementary Fig. 1, 2). There was no significant difference in review period according to the therapeutic area (Supplementary Fig. 5).

### Effect of GMP on New Drug Review Period

In Korea, NDA reviews comprise parallel reviews of multiple aspects, including safety and efficacy, specification and test methods, and GMP. It has been observed that the GMP review process takes longer on average than the safety and efficacy review process or specification and test methods review process. In other words, the GMP review process has a significant impact on determining the overall review period. The dataset has shown that the GMP review period has a significant impact on the overall review period for both chemical and biological drugs. And for biological drugs, the review period was even longer due to a delay in GMP review.

### Comparing Submission and Approval Gaps with US FDA and EU EMA

**Figure 4:**
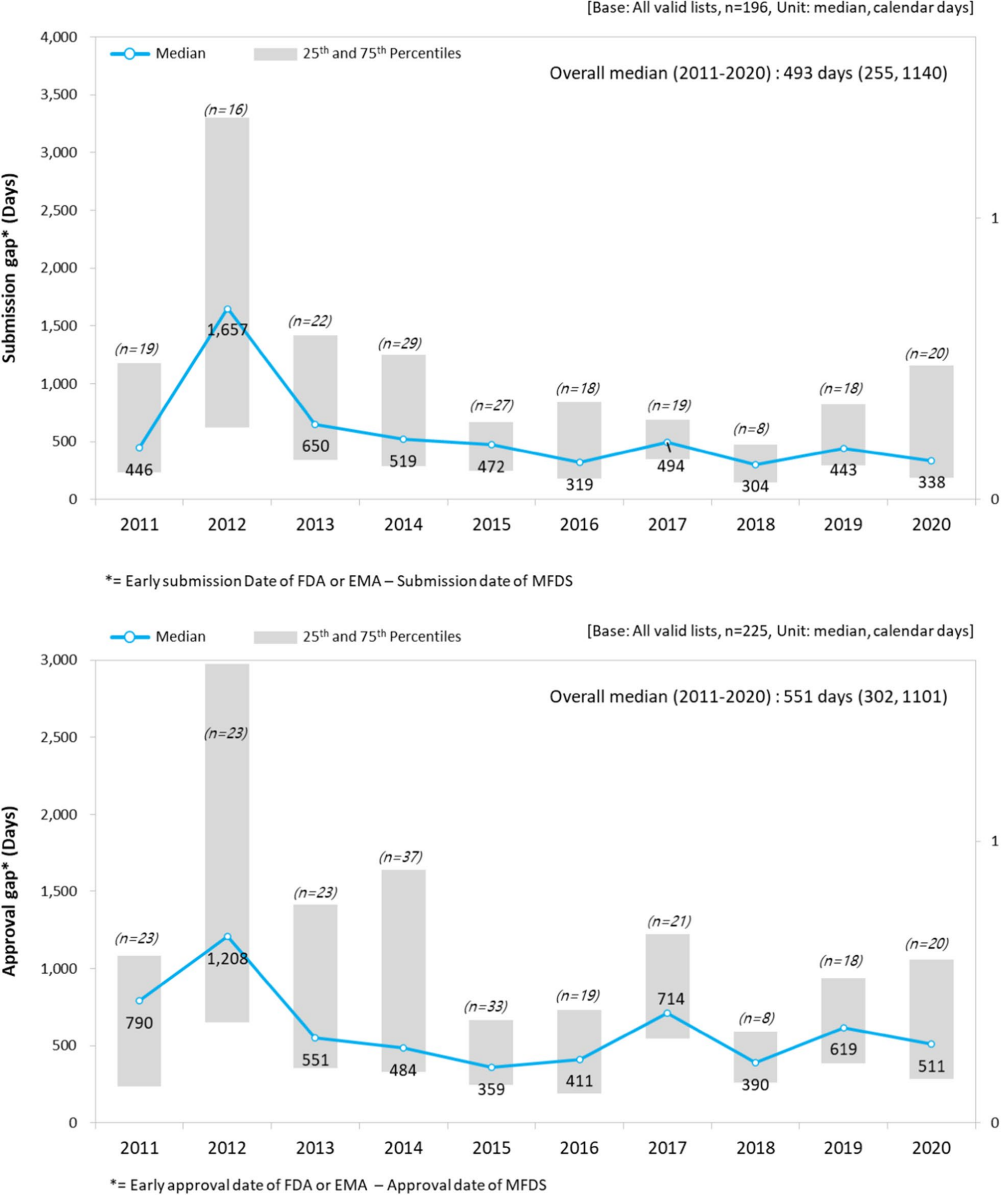
Submission and approval gaps between MFDS and FDA or EMA.

It was observed that all new drugs submitted for approval in Korea from 2011 to 2020 were submitted for review later than in the US or Europe, with an average difference of approximately 493 days (median) (Fig. 4).

Meanwhile, when the approval gap, which compares the date a new drug was approved in the US or Europe with the date the new drug was approved in Korea, was analyzed, a difference of approximately 551 days (median) was found (Fig. 4). This implies that a new drug was approved in Korea 551 days after it was approved in the US or Europe.

## Discussion and Conclusion

We observed that the delay in the NDA review time-frame increased from 2011 to 2020 in Korea. The median review time of the 235 study drugs submitted for NDA review was 315 days. The delay increased by an average of 15.4 days, from 276 days in 2011 to 353 days in 2020 (*P* < 0.0001). Among these new drugs, 166 were chemical drugs (70.6%) and 69 were biological drugs (29.4%), with a median review period of 291 and 353 days, respectively. Biological drugs showed an average of 43.2 days longer approval timeline than that for chemical drugs (*P* = 0.0054). All new drugs approved in Korea were submitted for review later than those in the US and EU, which resulted in an approximately 493-day median submission gap and a 551-day median approval gap, indicating that there is significant drug approval lag in Korea when compared to the US and EU.

A prior study investigated the delay in approval of new drugs in Korea and Japan, and the approval lag for 158 new drugs approved in Korea from 2009 to 2017 was 28.2 months [12]. The original approval date, type of product, and therapeutic class were analyzed as factors affecting drug lag, but there were no studies on the review period. When compared with the precedent study, this study confirmed that the drug lag of new drugs in Korea was significantly lower, from 28.2 to 18.4 months (551 days) (prior study period vs. this study period: 2009–2017 vs. 2011–2020) [12, 13].

Our study included 88 orphan drugs (37.4% of the study drugs) with an average review timeline of 130.4 days, which were approved faster than other non-orphan drugs (*P* < 0.0001). However, from 2016, the NDA review time-frame of orphan drugs became similar or even longer than that of non-orphan drugs, despite the enactment of ‘Rare Disease Management Act’ in 2016 which was aimed at promoting the development of orphan drugs and to improve patient access. In 2018 and 2019, NDA review times were longer for some orphan drugs than those of non-orphan drugs. This change may be due to the revision of the Pharmaceutical Affairs Act in August 2014. Prior to the revision, GMP inspection was exempted for orphan drugs. However, the GMP inspection exemption clause for orphan drugs was deleted in the revision, and thus, the review period for orphan drugs has gradually increased over time.

We found that the GMP review process had a significant influence on the overall review period for both chemical and biological drugs. The GMP review process took longer than the safety and efficacy review, specifications, and test method review periods. A delay in GMP review can occur when an on-site inspection is required, and the scheduling of inspection and the timeline to obtain approval may have contributed to the delay. The GMP review period has a significant impact on the overall review period for both chemical and biological drugs. The review period also showed differences among the therapeutic areas. However, the difference was not statistically significant. There are analysis of the GMP review period for chemical drugs and biological drugs in the supplementary Fig. 3, 4.

This study provides evidence for the review period of new drugs in Korea using data from multinational pharmaceutical companies. However, it was difficult to obtain information on whether the various policies and tools introduced to improve the review period affected the actual new drug review and approval timelines. As in Japan, additional research is needed to determine the impact of the policies and tools to increase the accessibility of new drugs [14–23].

It is important to use relevant laws to improve the accessibility of new drugs. However, a more practical and careful implementation of the law is required by regulatory authorities. It can be concluded that it is essential for the government and industry to make continuous efforts to systemically streamline the review period and system and ultimately reduce drug lag for new drugs.

In conclusion, despite some efforts to streamline the review system, the NDA review period has steadily increased by 15.4 days over the last 10 years in Korea. Particularly, after the enactment of the ‘Rare Disease Management Act’ in 2016, the delay in orphan drug reviews has further increased. Further research is necessary to identify the factors associated with the delayed review period of new drugs and to develop policies and regulations that can accelerate new drug approval.

In the FDA and EMA, the expedited review process accelerated the approval of new drugs when compared to the standard review process. MFDS established the Expedited Review Division in August 2020. We anticipate that the expedited review system will shorten the review period for the approval of new drugs in Korea as well.

## Supplementary Information

Below is the link to the electronic supplementary material.

Supplementary file1 (DOCX 356 kb)

## Data Availability

The data that support the findings of this study are available from the Korean Research-based Pharmaceutical Industry Association (KRPIA) of South Korea but restrictions apply to the availability of these data due to domestic laws and regulations that prohibit the distribution or release of companies' data to the public, and so are not publicly available. Data are however available from the authors upon reasonable request and with permission of the KRPIA of South Korea. But some data are available as open sources from the Ministry of Food and Drug Safety of South Korea so it can release to the public.
