# Supplementary material for: Changes in the Review Period of Drug Application and a Drug Lag from the FDA and the EMA: An Industry Survey in South Korea Between 2011 and 2020
Source: Ther Innov Regul Sci. 2022 Dec 20;57(3):552–60. doi: 10.1007/s43441-022-00486-x (PMC10133380; doi:10.1007/s43441-022-00486-x)
Supplement: Supplementary file 1 — Supplementary file1 (DOCX 356 kb) [file 43441_2022_486_MOESM1_ESM.docx]

**Supplementary materials**


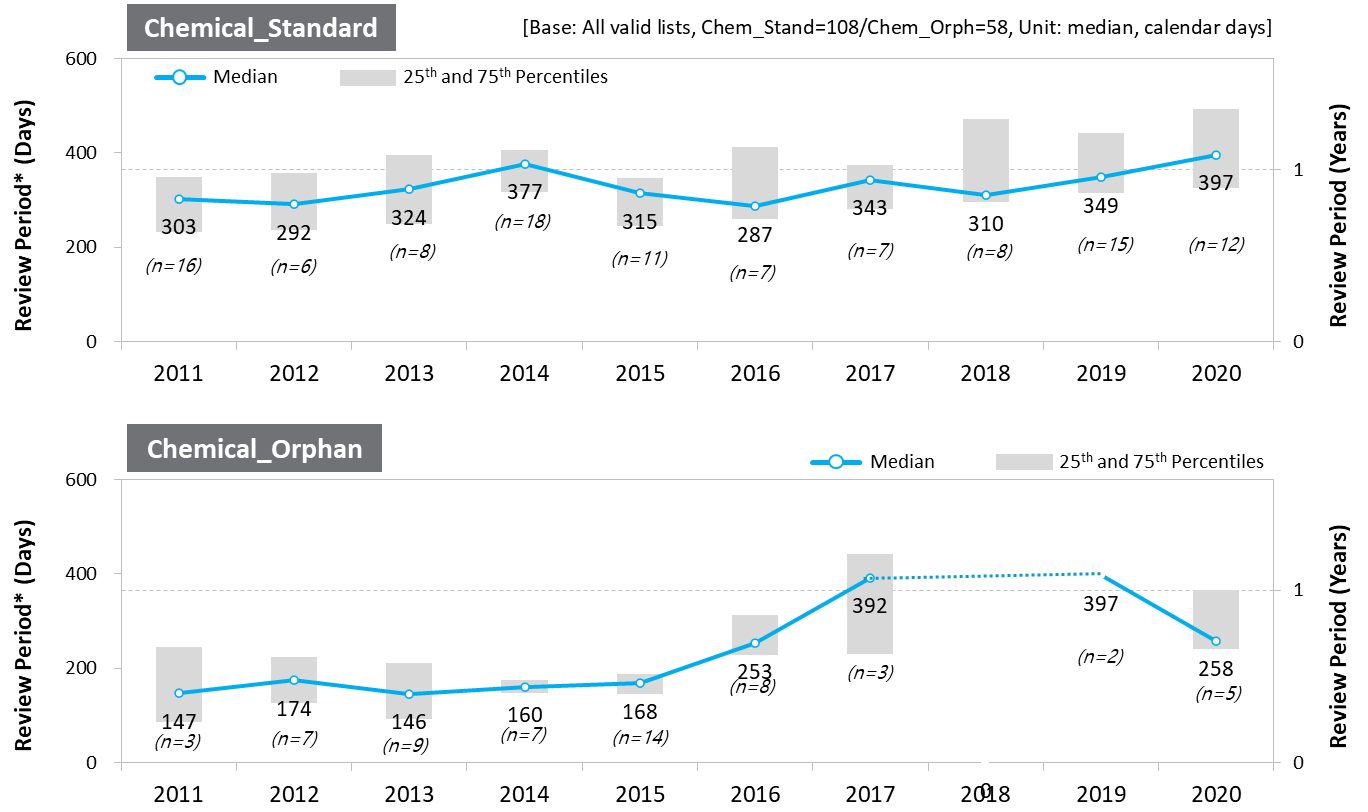


Supplementary figure 1. [Chemical] Standard/Orphan NDA Review Period


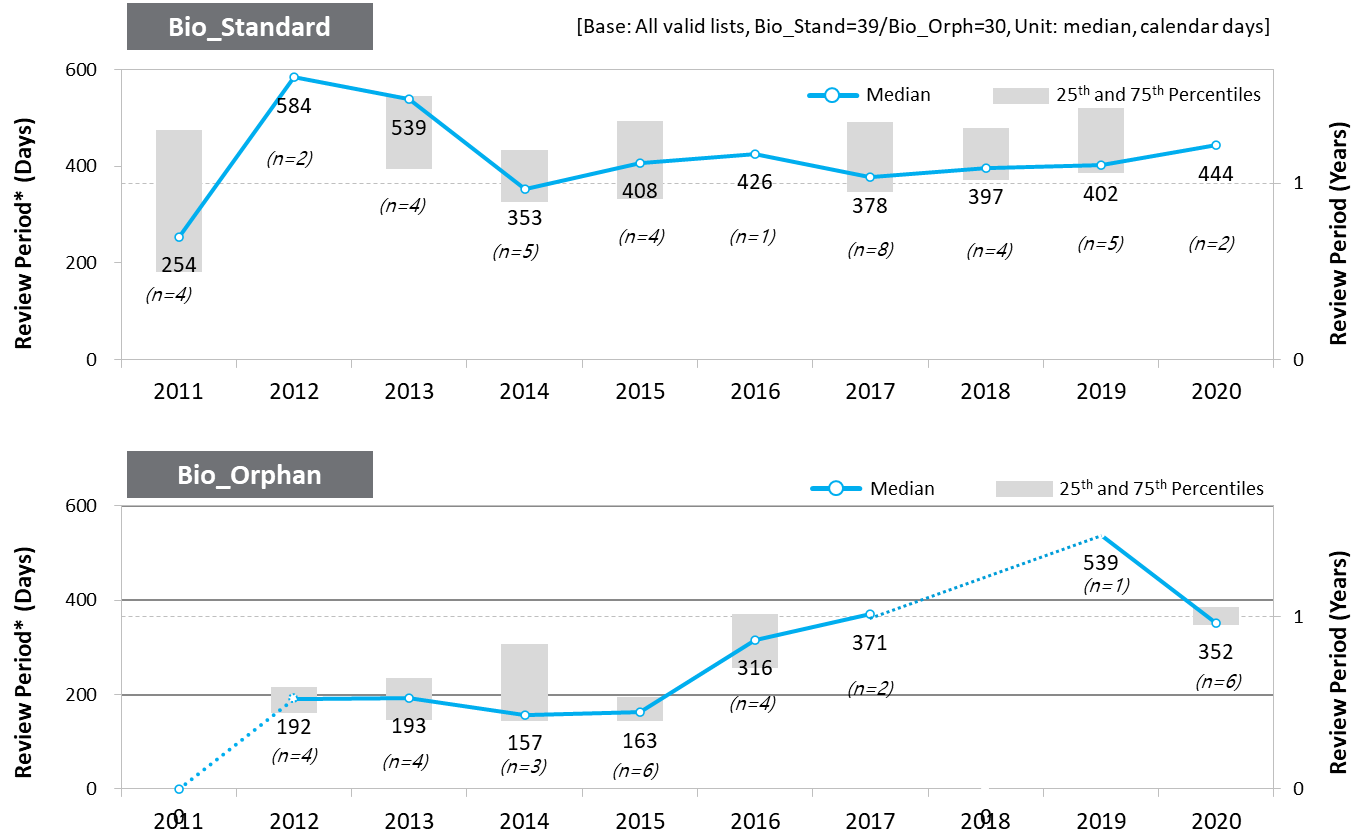


Supplementary figure 2. [Bio] Standard/Orphan NASs Review Period


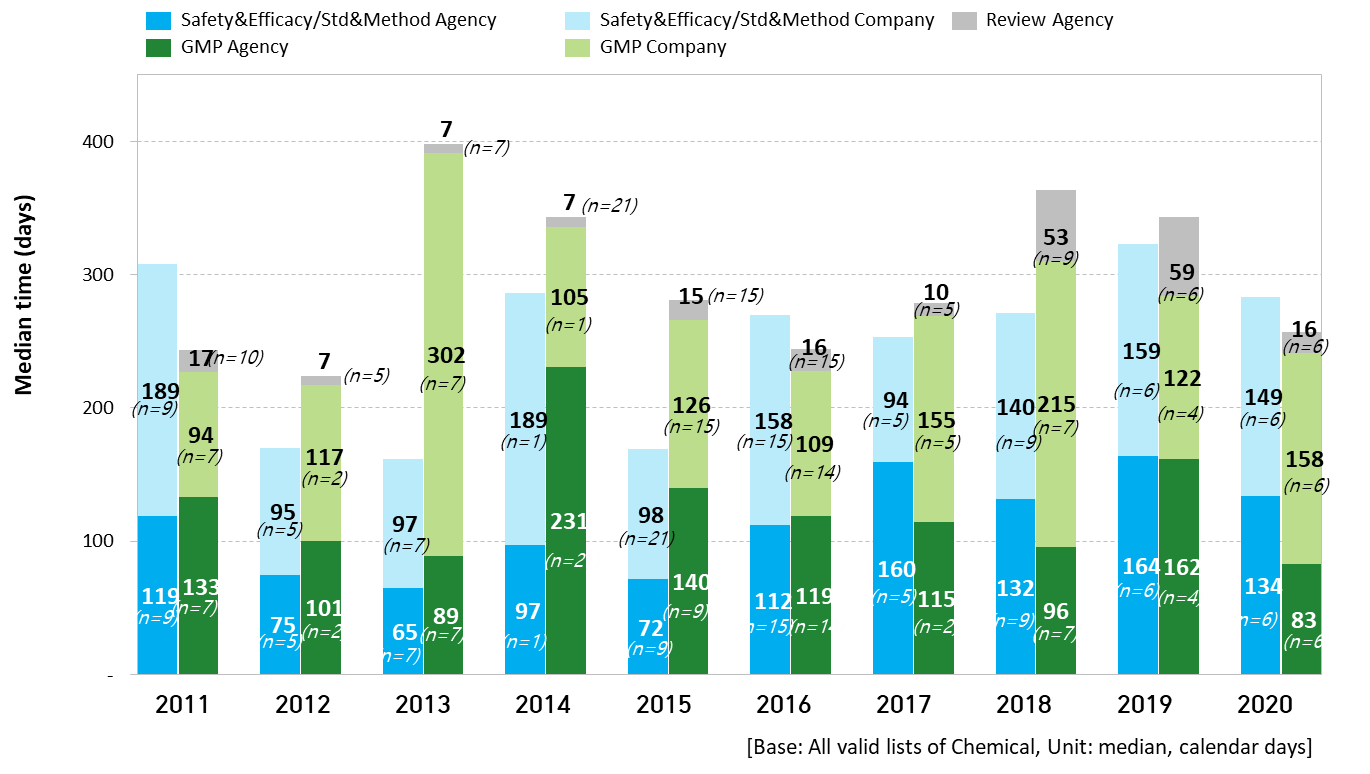


Supplementary figure 3. [Chemical] Safety-Efficacy review/Standard and Method review/GMP review period


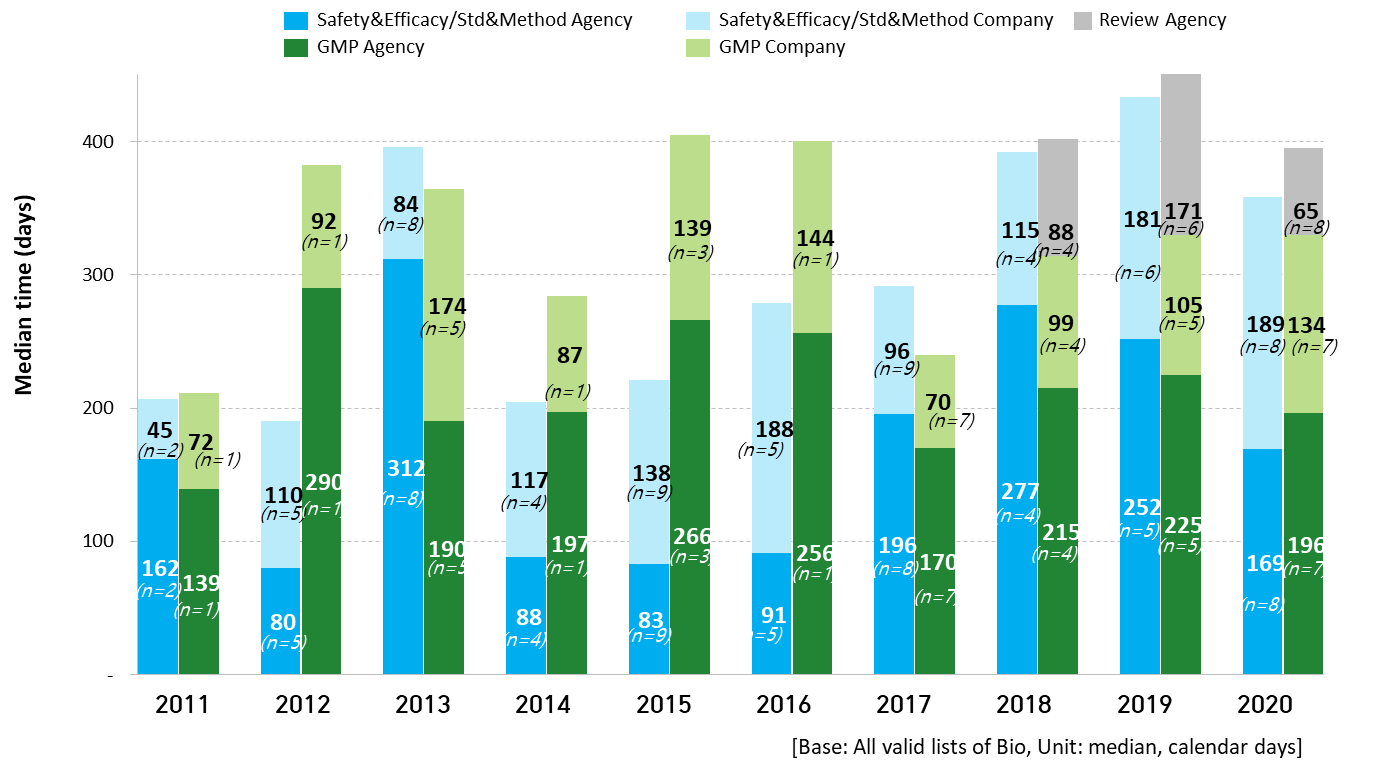


Supplementary figure 4. [Bio] Safety-Efficacy review/Standard and Method review/GMP review period


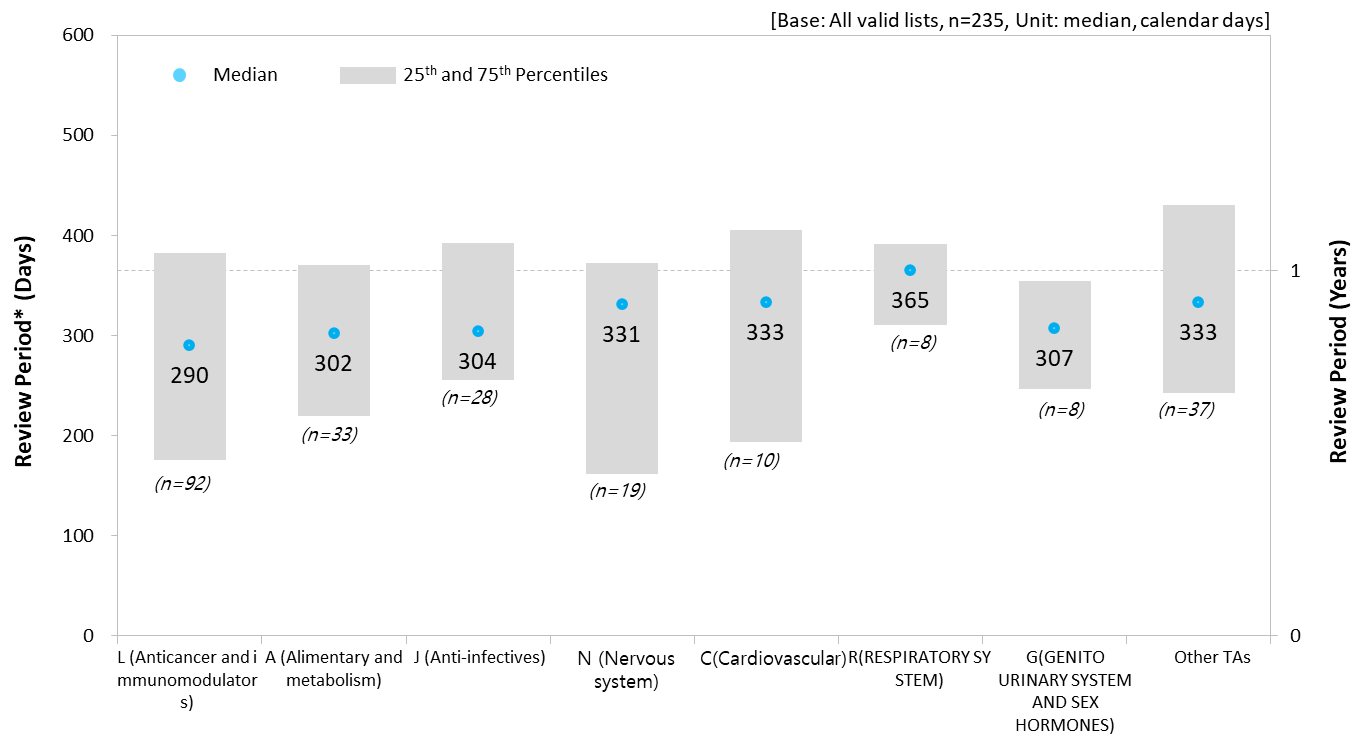
 Supplementary figure 5. NDA Review Period by TA
